# Chemical Defense Balanced by Sequestration and *De Novo* Biosynthesis in a Lepidopteran Specialist

**DOI:** 10.1371/journal.pone.0108745

**Published:** 2014-10-09

**Authors:** Joel Fürstenberg-Hägg, Mika Zagrobelny, Kirsten Jørgensen, Heiko Vogel, Birger Lindberg Møller, Søren Bak

**Affiliations:** 1 Plant Biochemistry Laboratory, Department of Plant and Environmental Sciences, Copenhagen Plant Science Centre, University of Copenhagen, Copenhagen, Denmark; 2 VILLUM Research Center “Plant Plasticity”, Department of Plant and Environmental Sciences, University of Copenhagen, Copenhagen, Denmark; 3 Department of Entomology, Max Planck Institute for Chemical Ecology, Jena, Germany; University of Heidelberg, Germany

## Abstract

The evolution of sequestration (uptake and accumulation) relative to *de novo* biosynthesis of chemical defense compounds is poorly understood, as is the interplay between these two strategies. The Burnet moth *Zygaena filipendulae* (Lepidoptera) and its food-plant *Lotus corniculatus* (Fabaceae) poses an exemplary case study of these questions, as *Z. filipendulae* belongs to the only insect family known to both *de novo* biosynthesize and sequester the same defense compounds directly from its food-plant. *Z. filipendulae* and *L. corniculatus* both contain the two cyanogenic glucosides linamarin and lotaustralin, which are defense compounds that can be hydrolyzed to liberate toxic hydrogen cyanide. The overall amounts and ratios of linamarin and lotaustralin in *Z. filipendulae* are tightly regulated, and only to a low extent reflect the ratio in the ingested food-plant. We demonstrate that *Z. filipendulae* adjusts the *de novo* biosynthesis of CNglcs by regulation at both the transcriptional and protein level depending on food plant composition. Ultimately this ensures that the larva saves energy and nitrogen while maintaining an effective defense system to fend off predators. By using *in situ* PCR and immunolocalization, the biosynthetic pathway was resolved to the larval fat body and integument, which infers rapid replenishment of defense compounds following an encounter with a predator. Our study supports the hypothesis that *de novo* biosynthesis of CNglcs in *Z. filipendulae* preceded the ability to sequester, and facilitated a food-plant switch to cyanogenic plants, after which sequestration could evolve. Preservation of *de novo* biosynthesis allows fine-tuning of the amount and composition of CNglcs in *Z. filipendulae*.

## Introduction

A driving force in speciation is to evolve, exploit and handle chemical defenses. Two central issues with regards to insect chemical defenses are how cost-efficient defenses against predators have evolved and how these are regulated. Insects acquire chemical defense compounds either through *de novo* biosynthesis, sequestration (uptake, accumulation and storage [1] of plant-derived defense compounds) or by a combination of the two strategies [2]. *De novo* biosynthesis requires maintenance of a biosynthetic machinery as well as use resources that could otherwise be allocated to growth and development, but provides the possibility to tightly regulate the composition and amounts of defense compounds. Sequestration relies on the composition of the compounds in the food-plant and thus enables less control by the insect, but is a more cost-efficient strategy since the compounds do not have to be produced *de novo*. While some specialized leaf beetle (Chrysomelinae) species have evolved to biosynthesize defense compounds *de novo*, others sequester them from plants and metabolize them to generate new defense compounds [2]. Similarly, within butterflies (Heliconiini) there are species that either sequester or biosynthesize defense compounds *de novo* [3], [4], [5]. In contrast, the larvae of Zygaena moths (Zygaenoidae, Lepidoptera), are unique in that they can obtain the same chemical defense compounds through both *de novo* biosynthesis [4] and sequestration [6].

**Figure 1:**
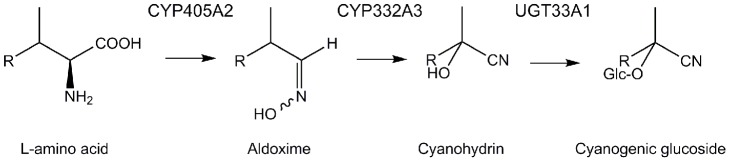
Biosynthesis of cyanogenic glucosides in *Z. filipendulae*. R represents a methyl group in linamarin and an ethyl group in lotaustralin.

Plants produce an astonishing array of bioactive specialized compounds in the warfare against herbivores [7]. Cyanogenic glucosides (CNglcs) are one class of such compounds and they are present in more than 2650 higher plant species. They are generally regarded as feeding deterrents against herbivores due to their bitter taste, but they can also be bioactived by plant β-glucosidase-mediated hydrolysis which leads to release of toxic hydrogen cyanide (HCN) [8]. Although highly efficient against generalist insect herbivores, many specialist insects have evolved mechanisms to tolerate CNglcs and some may even utilize them as phagostimulants and/or oviposition cues [9], [10], [11]. The Burnet moth *Zygaena filipendulae* contains the CNglcs linamarin and lotaustralin [12], that are used for defense against predators [13], [14], [15]. These CNglcs can be sequestered by the larva from its food-plant *Lotus corniculatus* (Fabaceae) [6] and also biosynthesized *de novo* from the amino acids valine (Val) and isoleucine (Ile), respectively [4]. The biosynthetic pathways in *Z. filipendulae* and *Lotus japonicus* involves the same intermediates and enzymatic steps [16], but analysis of the encoding genes revealed that the pathway for CNglc biosynthesis have evolved convergently in these two Kingdoms of Life [17]. The *de novo* biosynthesis is catalyzed by two multifunctional cytochromes P450 (P450s) and a UDP-glycosyltransferase (UGT) in both *L. japonicus* and *Z. filipendulae* [16], [17], [18]. The *Z. filipendulae* enzymes are CYP405A2, CYP332A3 and UGT33A1, respectively (Figure 1) [17].

**Figure 2:**
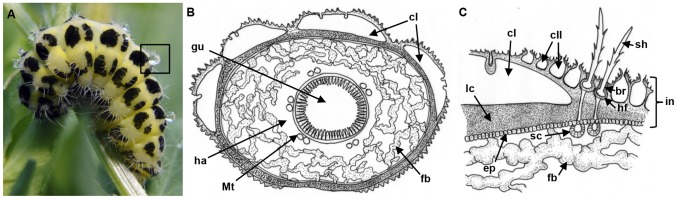
*Z. filipendulae* larval anatomy. A) Larva releasing defense droplets, containing CNglcs, from large (type I) cuticular cavities following physical irritation. B) Graphical representation of cross section of fifth instar larva. C) Close-up of larval integument with cavities and hairs. br, basal ring; cI, large (type I) cuticular cavity; cII, small (type II) cuticular cavity; ep, epidermis; fb, fat body; gu, gut; ha, haemolymph; hf, hair follicle; in, integument; lc, lamellate cuticle; Mt, Malpighian tubules; sc, sensory cell; and sh, sensory hair (seta). Drawings were made by Kirsten Lehrmann Madsen.

*Z. filipendulae* larvae accumulate CNglcs mostly in the haemolymph and cuticular cavities in the integument (Figure 2 and Figure S1) [19]. Upon physical irritation, larval muscle contraction leads to CNglc secretion from two types of cavities in the form of sticky droplets (Figure 2A). The larger (type I) cavities are situated below the black pigmented patches on the larval body and react to slight physical irritation, while the smaller (type II) cavities requires a stronger irritation before they are emptied. The defense droplets are known to have a deterrent effect against predators, such as ants, frogs and birds [13].

The overall level and ratio between linamarin and lotaustralin has been shown to be tightly regulated in *Z. filipendulae* [11], probably due to the important roles these compounds play during the insect life-cycle [20]. They have been shown to take part in male-female communication, be part of a nuptial gift from the male to the female, and may also be involved in nitrogen metabolism [21]. Accordingly, *Z. filipendulae* larvae feeding on *L. corniculatus* lacking CNglcs (acyanogenic, [(−)diet]) show similar overall CNglc level and ratio of linamarin to lotaustralin as larvae feeding on cyanogenic plants [(+)diet], suggesting a fine-tuned ability to balance the *de novo* biosynthesis in response to the CNglc content of the food-plant [11]. It is not known, however, why the ratio between linamarin and lotaustralin is important, since they have very similar structures, differing only by a methyl group. Non-choice feeding studies show that *Z. filipendulae* larvae reared on (−)diet experience reduced growth, lower body mass at pupation and higher mortality compared to larvae reared on (+)diet [11]. In addition, free-choice feeding studies have shown that *Z. filipendulae* larvae preferentially feed on (+)diet [11]. Thus, *de novo* biosynthesis is associated with an apparent higher cost or reduced fitness, as compared to sequestration from the food-plant. Yet *de novo* biosynthesis has been preserved in evolution.

Here, we report how *Z. filipendulae* larvae alternate between *de novo* biosynthesis and sequestration based on the ingested amount of plant-derived CNglcs in the food-plant to secure homeostasis in their chemical defense. Contrary to earlier assumptions that hypothesized biosynthesis in the inner organs of the larva [22], we demonstrate that *de novo* biosynthesis takes place in organs and cell types associated with the integument. This localization infers the possibility of a rapid transport of newly biosynthesized CNglcs into the haemolymph and cuticular cavities for storage. We demonstrate that *Z. filipendulae* regulates CNglc biosynthesis, both at the transcript and protein level, as a response to the CNglc level in the food-plant and thereby probably optimizes resource allocation. Finally, our data supports the hypothesis that *de novo* biosynthesis of CNglcs in *Zygaenidae* preceded the ability to sequester, thereby facilitating colonization and subsequently uptake of CNglcs from their food-plant.

## Results

### *De novo* biosynthesis of CNglcs in larvae is dependent on the CNglc content of the food-plant

**Figure 3:**
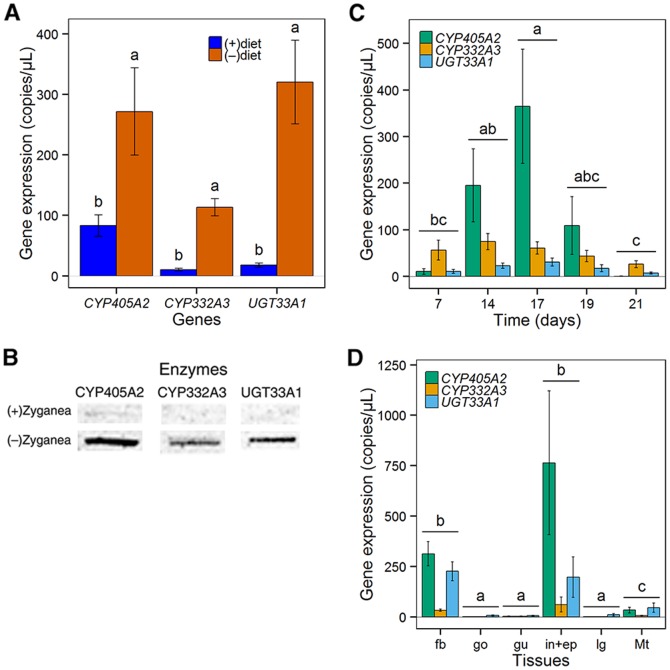
Cyanogenic glucoside biosynthesis in *Z. filipendulae* larvae dependent on diet. A) Transcript levels in seventh instar larvae reared on (+)diet are lower compared to larvae reared on (−)diet respectively (MannWhitney *U* = 6343, *P*<0.001). Means ± SEM are shown with number of replicates equal to 15 for female and 15 for male larvae. Statistical differences between diets are indicated by different letters. B) Western blot comparing enzyme levels in seventh instar larvae reared on (+)diet and (−)diet, respectively, with number of replicates equal to 10 for each diet. Equal amounts of protein were applied, as related to larval mass. C) Transcript levels in third instar larvae 7–21 days following transfer from (+)diet to (−)diet. Expression of *CYP405A2* varies over time (Kruskal-Wallis *H* = 18, df = 4, *P*<0.01). The *CYP405A2* transcript levels are significantly higher at day 14 compared to day 7 (Posthoc multiple comparison test, *P*<0.05) and day 21 (Posthoc multiple comparison test, *P*<0.05) respectively. The transcript levels of the other two biosynthetic genes do not differ significantly between the days. Means ± SEM are shown with number of replicates equal to 8 at day 7–19 and 6 at day 21. Statistical differences between days are indicated by different letters and overlapping expression levels between days are indicated with multiple letters where overlapping groups share at least one letter. D) Transcript levels vary between tissues of seventh instar larvae reared on (+)diet or (−)diet (Kruskal-Wallis *H* = 142, n_fb_ = n_gu_ = n_in+ep_ = n_lg_ = n_Mt_ = 21, df = 5, *P*<0.001). No significant difference is observed between fat body, integument and epidermis, while the transcript levels in these tissues are significantly higher than the remaining tissues (Posthoc multiple comparison test, *P*<0.001). The transcript levels of the Malphigian tubules are also significantly higher than the gonads, gut and labial glands (Posthoc multiple comparison test, *P*<0.001). fb, fat body; go, gonads (♀); gu, gut; in, integument; lg, labial glands; and Mt, Malpighian tubules. Means ± SEM are shown with number of replicates equal to 7 of each tissue. Statistical differences between tissues are indicated by different letters.

Previous studies show that *Z. filipendulae* larvae prefer feeding on (+)diet, and that feeding on (−)diet results in growth reduction. In order to test if the larvae regulate the *de novo* biosynthesis of CNglcs in relation to CNglc content of the food plant, transcript levels of the biosynthetic genes were analyzed by quantitative real-time PCR (qRT-PCR) and RNA-Seq in seventh instar larvae collected in the field and then reared for 2 weeks on (+)diet and (−)diet respectively. The gene expression in males and females was first analyzed separately, but since there was no significant difference in gene expression between the two sexes, male and female larvae were examined jointly. The qRT-PCR analyses revealed that larvae reared on (−)diet expressed the three biosynthetic genes at levels more than threefold higher compared to larvae reared on (+)diet (MannWhitney *U* = 6343, n_(−)diet_ = n_(+)diet_ = 15, *P*<0.001; Figure 3A). The same result was obtained by RNA Seq which showed 3.2, 3.8 and 1.9 fold higher expression of *CYP405A2*, *CYP332A3* and *UGT33A1*, respectively, between larvae reared on (−)diet and (+)diet. *CYP405A2* expression tended to be higher than that of *CYP332A3*, which is consistent throughout the transcriptional analyses, whereas expression of *UGT33A1* relative to the other genes is less consistent between the experiments. The larvae were collected in the field, and the large standard error of the mean observed for each gene and diet is most likely caused by the non-synchronized larval growth and development.

Antibodies raised against the three biosynthetic enzymes (Figure S2) were used to compare the levels of the individual biosynthetic enzymes in larvae reared on (−)diet and (+)diet respectively by Western blotting. High biosynthetic enzyme levels could only be detected in the larvae reared on (−)diet (Figure 3B). CYP332A3 and UGT33A1 were not detected in larvae reared on (+)diet, and only a very faint band corresponding to CYP405A2 could be detected. This is in agreement with the qRT-PCR data that demonstrate a basal level of the *CYP405A2* transcript even in the larvae reared on (+)diet, and shows that regulation of *de novo* biosynthesis of CNglcs is manifested on the transcription as well as the protein level.

### *CYP405A2* controls the flux into CNglc biosynthesis and is regulated at both the transcript and protein level

To follow the regulation of the pathway over time, third instar larvae hatched in the laboratory and reared on (+)diet were transferred to and reared on (−)diet for up to 3 weeks, and the expression of the three biosynthetic genes was analyzed by qRT-PCR. *CYP405A2* encodes the enzyme that catalyzes the first step in the CNglc biosynthetic pathway, the transformation of the amino acids into the corresponding oximes. The expression of *CYP405A2* varied over time (Kruskal-Wallis *H* = 18, n_7_ = n_14_ = n_17_ = n_19_ = 8, n_21_ = 6, df = 4, *P*<0.01). At day 7 following transfer to (−)diet, the expression of *CYP405A2* had only reached basal levels. *CYP405A2* expression then increased, following a bell shaped curve with the highest expression at day 17, which was significantly higher than at day 7 (Posthoc multiple comparison test, *P*<0.05), after which basal levels were detected again at day 21, also significantly lower than at day 17 (Posthoc multiple comparison test, *P*<0.05; Figure 3C). In contrast, *CYP332A3* and *UGT33A1* exhibited more constant and much lower expression levels than observed for *CYP405A2*, except for day 7 and 21, when they were more highly expressed than *CYP405A2*. The highly time dependent expression levels imply that *CYP405A2* encodes the enzyme that regulates the flux into the pathway.

### The fat body and cells associated with the integument constitute the main sites of *de novo* biosynthesis of CNglcs in larvae

**Figure 4:**
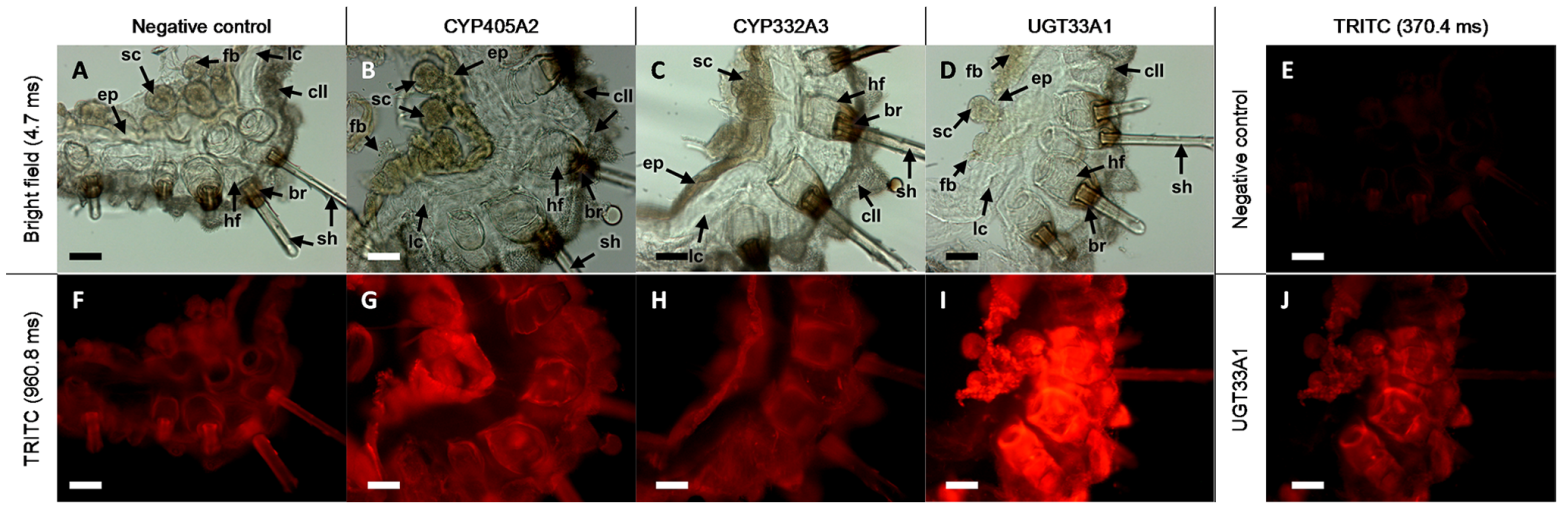
Tissue specific cyanogenic glucoside biosynthetic gene expression in *Z. filipendulae* larvae. Tissue localization of *CYP405A2*, *CYP332A3* and *UGT33A1* in a representative larva determined by *in tube in situ* PCR analysis on 80 µm transverse sections using tetramethylrhodamine isothiocyanate (TRITC) and analyzed using fluorescence microscopy. A–D) Controls to visualize the different cell types analyzed using light microscopy, with 4.7 ms exposure time. E) Negative control excluding primers in the PCR reaction, visualized with red light excitation and 370.4 ms exposure time. F) Negative control visualized with red light excitation and 960.8 ms exposure time. G–I) Expression of CNglc biosynthetic genes as monitored by TRITC labeling, visualized with red light excitation and 960.8 ms exposure time. J) Expression of *UGT33A1* as monitored by TRITC labeling, visualized with red light excitation and at 370.4 ms exposure time. br, basal ring; cII, small (type II) cuticular cavity; ep, epidermis; fb, fat body; hf, hair follicle; lc, lamellate cuticle; sc, sensory cell; and sh, sensory hair (seta). Scale bars: 100 µm.

To identify the site(s) of *de novo* biosynthesis in *Z. filipendulae* larvae, transcript levels of the biosynthetic genes were analyzed in dissected larval tissues using qRT-PCR as well as thin larval transverse sections by *in tube in situ* PCR. qRT-PCR revealed tissue specific expression patterns (Kruskal-Wallis *H* = 142, n_fb_ = n_gu_ = n_in+ep_ = n_lg_ = n_Mt_ = 21, df = 5, *P*<0.001), with high expression in the fat body and the integument including the epidermis, as well as low expression in the Malpighian tubules (Figure 3D). There were no significant quantitative differences between the expression in the fat body and the integument. The expression in those tissues was significantly higher than that observed in the Malpighian tubules (Posthoc multiple comparison test, *P*<0.001). The expression in the Malpighian tubules was also significantly higher than the remaining tissues (Posthoc multiple comparison test, *P*<0.001). Similarly to what we previously observed in intact larvae (Figure 3A), there was a tendency of higher expression of *CYP405A2* and *UGT33A1* compared to *CYP332A3* (Kruskal-Wallis *H* = 13, n_fb_ = n_gu_ = n_in+ep_ = n_lg_ = n_Mt_ = 7, df = 2, *P*<0.005), although only the latter difference was statistically significant (Posthoc multiple comparison test, *P*<0.001). Using *in tube in situ* PCR the biosynthetic gene transcripts were amplified and labeled with tetramethylrhodamine isothiocyanate (TRITC), and visualized by fluorescence microcopy, which enabled detection at the tissue level. However, since *in tube in situ* PCR cannot be used for quantification of gene expression, the differences in intensity must be taken with caution. The fluorescence of *CYP405A2* was highest within the epidermis, the fat body and sensory cells, followed by the cuticular cavities and the edge of the sensory hair follicles (Figure 4B and G as well as Figure S3B). *CYP332A3* showed the most intense fluorescence in the epidermis (Figure 4C and H as well as Figure S3C). In contrast to the two P450 transcripts, the fluorescent pattern of the glycosyltransferase *UGT33A1* was less confined, and a strong fluorescent signal was observed in the sensory hair follicles, the epidermis, the fat body, the cuticular cavities and the sensory cells (Figure 4D, I and J). No fluorescent labeling was detected in the lamellate cuticle.

**Figure 5:**
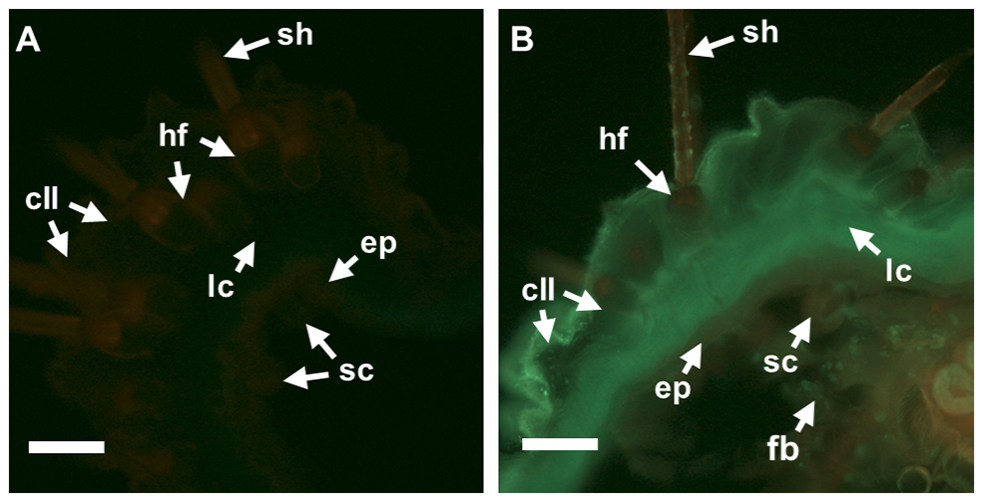
Tissue specific localization of the cyanogenic glucoside biosynthetic pathway in *Z. filipendulae* larvae. Tissue localization of CYP405A2 in a representative larva determined by immunolocalization on 100 µm transverse larval sections using fluorescein isothiocyanate (FITC) labeled polyclonal antibodies and analyzed using fluorescence microscopy. A) Background control labeling with FITC as observed without primary antibodies. B) Polyclonal antibodies raised against CYP405A2. cII, small (type II) cuticular cavity; ep, epidermis; fb, fat body; hf, hair follicle; sc, sensory cell; and sh, sensory hair (seta). Scale bars: 100 µm.

The antibodies raised against CYP405A2 peptides were used for localization studies in thin transverse larval sections. CYP405A2 was found to be situated in the lamellate cuticle, which is in contrast to the localization of the corresponding gene transcripts (Figure 4G), as well as the cuticular cavities, the fat body, the epidermis and sensory cells (Figure 5) in agreement with the *in tube in situ* PCR results. Unfortunately, antibodies raised against CYP332A2 and UGT33A1 peptides did not have the monospecificity required for immunolabeling studies using larval sections.

## Discussion

Cyanogenesis, the liberation of HCN from CNglcs, is polymorphic in some plant species. Consequently, the level and ratios of linamarin and lotaustralin as well as the quantity of the degradation enzymes vary widely, as observed in e.g. *L. corniculatus* and *Trifolium* spp. (clover) [23], [24], [25]. Polymorphism in cyanogenesis has been observed among individual plants of the same species, in response to environmental challenges and during plant ontogeny [24], [26], [27], [28], [29]. It enables a plant population to have a more plastic response required to cope with both generalist and specialist herbivores. Plants with high cyanogenesis show increased resistance against generalist herbivores and the defense is thus especially effective in years when such herbivores are abundant [30], [31]. On the other hand, highly cyanogenic plants are more sensitive to frost and drought [31] and also preferred by specialist herbivores, such as *Z. filipendulae* [11]. Although *Z. filipendulae* larvae are seldom expected to feed on completely acyanogenic plants *in vivo*, and thereby to rely solely on *de novo* biosynthesis, the larvae would need to be able to adjust their CNglc levels due to the polymorphism in cyanogenesis in *L. corniculatus* populations, in order to maintain the optimal content and ratio of CNglcs during their life-cycle [11]. Sequestration seem to be playing the dominant role in CNglc acquisition, probably because it is associated with fewer energetic costs and thus results in increased fitness of the insects [11]. This study demonstrates that *de novo* biosynthesis of CNglcs in *Z. filipendulae* larvae is only dominant when required, and implies that the ability to *de novo* biosynthesize CNglcs probably complements sequestration in order to compensate for differences in turnover of linamarin and lotaustralin to maintain CNglc homeostasis [11].

The detailed expression analyses and immunolocalization studies demonstrate that *de novo* biosynthesis of CNglcs is mainly localized to the integument and the neighboring fat body. The fat body is a major site of fat storage and a central organ for integration and coordination of hormonal and nutritional signals regulating insect metamorphosis. In addition, the fat body is the site of synthesis of most haemolymph proteins as well as for circulating metabolites [32]. Our findings are in contrast to an earlier study in the closely related species *Zygaena trifolii* [33]. In that study, it was suggested that *de novo* biosynthesis of CNglcs takes place within organs such as the gut, fat body or haemolymph, while the epidermis is mainly involved in transport and accumulation of CNglcs within the cuticular cavities. Due to the apparent lack of cuticular ducts through the cuticle into the cavities or special morphological adaptations for secretion, CNglcs were further hypothesized to be transported into the cavities when these were formed in the course of the molting process [19]. However, a recent study demonstrates transport of CNglcs into the cavities in non-molting larvae [34]. This implies an alternative transportation mechanism in non-molting larvae. In addition, the data presented in the current study are supported by our earlier proteomic and enzyme activity and localization studies which showed biosynthetic enzyme activity in the integumental microsomal fraction from *Z. filipendulae* [17], [20]. Taking all data into consideration, *de novo* biosynthesis of CNglcs most likely takes place both in the integument and the fat body. These sites of *de novo* biosynthesis infer a direct and continuous transport of CNglcs into the cuticular cavities as well as transport across the epidermis and from the fat body into the haemolymph. Since the transport proceeds against a concentration gradient (haemolymph: 0.05 mmol/ml, defensive fluid: 0.3 mmol/ml [35]), highly specific active transporters must be involved. An example is ABC-transporters that mediate the transport of the first intermediates in iridoid monoterpene *de novo* biosynthesis from the fat body in some leaf beetles (Chrysomelinae), to dorsal glands [36], [37], [38], [39], [40].

Some leaf beetles biosynthesize iridoid monoterpenes *de novo* from mevalonic acid whereas others produce them from precursors sequestered from their food-plants [41], [42]. An intermediate in the biosynthesis of the iridoid monoterpenes, 8-hydroxygeraniol, is an allosteric competitive inhibitor of the conversion of 3-hydroxy-3-methylglutaryl-coenzyme A (HMG-CoA) into mevalonic acid by HMG-CoA reductase (HMGR). Consequently, *de novo* biosynthesis is repressed when the larva is able to sequester iridoid precursors from the food-plant [43]. In contrast to the strategy employed by leaf beetles, *Z. filipendulae* regulates *de novo* biosynthesis of CNglcs at both the transcript and protein levels (Figure 3A and 3B).

When *Z. filipendulae* larvae were transferred from (+)diet to (−)diet, the transcript levels of the first gene in the pathway, *CYP405A2*, clearly changed over time, while the transcript levels of the other genes in the pathway appeared more constant. In addition, the expression of *CYP405A2* was lower compared to the two other genes at the beginning and the end of the time study. The expression patterns may reflect down-regulation of the biosynthetic pathway during molting, which in *Zygaena* living in the field occurs every 8–10 days [44], although development on (−)diet is always delayed compared to development on (+)diet (personal observations). Molting could thus potentially occur before and after the peak exhibited at day 17. The fluctuation in expression of *CYP405A2* over time indicates that CYP405A2 regulates the flux into the biosynthetic pathway, which is in agreement with it catalyzing the key signature step; the transformation of an amino acid to the corresponding oxime (Figure 1). Down-regulation of only *CYP405A2* would provide the larvae with a faster regulatory mechanism compared to down-regulation of the entire pathway. In plants, the signature step in a biosynthetic pathway for a specialized compound such as CNglcs is usually catalyzed by an enzyme with high substrate specificity [45], which would limit the number of substrates entering each particular pathway. Hence, the subsequent pathway enzymes may be more substrate promiscuous which would offer increased overall metabolic flexibility. CYP405A2 show higher preference for Ile than Val *in vitro* [17], which may serve to facilitate the suggested higher turnover of Ile-derived lotaustralin [46].

It is the general concept that *Zygaenidae* originally fed on acyanogenic plants belonging to the Celastraceae and was dependent on *de novo* biosynthesis of the two CNglcs linamarin and lotaustralin for defense [47]. *Zygaena* spp. later encountered and became adapted to cyanogenic plants [48] belonging to the Fabaceae, such as *L. corniculatus*. They could probably employ these as food plants because they already had the enzymatic machinery and possessed the necessary morphological adaptations to handle and store potentially toxic CNglcs. Similarly, *de novo* biosynthesis of saponins and cardenolides by leaf beetles (Chrysomelidae) [42], [49] and CNglcs by *Heliconius* butterflies (Papilionoidea) [50], [51] most likely facilitated colonization and subsequent sequestration from each of their respective food-plants. In analogy, we propose that the *de novo* CNglc biosynthetic pathway in *Z. filipendulae* was constitutively expressed before colonization of *L. corniculatus*. Thus, following the encounter with a cyanogenic food-plant, the larvae evolved the ability to regulate the pathway in dependence of the presence of CNglcs in the food-plant. By shifting from *de novo* biosynthesis to sequestration, the larva could probably preserve energy for primary metabolism and nitrogen for utilization in *e.g.* biosynthesis of the polymer chitin, a constituent of the integument [21]. Although it takes time before the larvae reach their full potential for *de novo* biosynthesis of CNglcs when transferred from (+)diet to (−)diet (Figure 3D), and the option of sequestration is less costly [52], we speculate that the capacity to biosynthesize CNglcs *de novo* has been preserved to enable counteraction of the polymorphism in *Lotus* plants of both the total amounts and the ratio between linamarin and lotaustralin. The fact that the CNglc pathway was not completely repressed on (+)diet is yet another indication that, although sequestration is the major contributor to the CNglc content in *Z. filipendulae* [34], *de novo* biosynthesis would be carried out too in order to fine-tune the total amount and ratio of linamarin and lotaustralin.

This study demonstrates that the biosynthetic pathway for CNglcs is constitutively expressed but repressed when sequestration is possible. The question then arises why all three biosynthetic genes are down-regulated and not just *CYP405A2*, the first gene in the pathway. One possibility is that CYP332A3 and UGT33A1 are involved in other processes that to some degree correlate with the repression of the biosynthetic pathway for CNglcs, making their overall expression pattern similar to each other. For instance in vertebrates, many P450s and UGTs are involved in detoxification reactions and are often co-regulated [53]. Indeed, based on proteomics data CYP332A3 and UGT33A1, but not CYP405A2, are also present in the major organs involved in detoxification, the excretory Malpighian tubules and the gut [17]. This could indicate that these two enzymes have an additional function in detoxification. The strong expression pattern of *UGT33A1* in multiple organs (Figure 3D and 4I) also agrees with a very diverse expression pattern of UGTs with regards to tissue specificity and ontogeny observed in insects in general [54], [55], [56], [57], [58], [59].

## Conclusions

We demonstrate that insects like *Zygaena* larvae react to the content and composition of chemical defense compounds in their food-plants. *De novo* biosynthesis compensates for differences in ratios and overall content of CNglcs in the food-plant, and thereby serves to complement sequestration. The biosynthetic sites of CNglcs in *Z. filipendulae* larvae are the fat body and the integument. Their close physical contact to the main storage sites of CNglcs makes it possible to have a rapid replenishment of the reserves of defense compounds following encounters with predators. Biosynthetic regulation is manifested at different levels, which provides a cost-efficient utilisation of resources. We propose that the biosynthetic pathway was originally constitutively expressed and that repression of the pathway evolved following a host-plant shift to cyanogenic plants, and subsequent contrivance of sequestration. Finally, additional functions for the two subsequent enzymes involved in CNglc biosynthesis are suggested. In conclusion, this study illuminates the intricate fine-tuning of *de novo* biosynthesis and sequestration of a chemical defense compound in insects, and it highlights the complex mechanisms that have evolved to optimize defense compounds while at the same time probably conserving energy for growth and development.

## Materials and Methods

### Ethics statement

No specific permissions were required for collecting *Z. filipendulae* larvae or *L. corniculatus* plants, as none of these species are endangered in Denmark. Authors maintained the population at sustainable levels.

### a) Insect rearing

Fifth to seventh instar *Z. filipendulae* larvae were collected from a natural population near Copenhagen (N 55°38.077′, E 12°15.748′) [11] and raised in the laboratory. Eggs laid in the laboratory later in the summer were collected, allowed to hatch and reared until diapause (fourth instar) in late summer. As *Z. filipendulae* larvae spend ∼8 months in diapause (∼September to April in the fourth instar), it was not feasible to maintain a continuous population in the laboratory. Larvae were reared in transparent plastic boxes, lined with tissue paper and supplied with new food plant material every 3 days. Two types of larval diets were used [11]: *L. corniculatus* lacking CNglcs [(−)diet]; and *L. corniculatus* producing CNglcs [(+)diet]. *Z. filipendulae* reared on (+)diet as well as *Z. filipendulae* reared on (−)diet contain equivalent total amounts and ratios of linamarin and lotaustralin (∼21 µg/mg fresh weight and a ∼1∶1 ratio of linamarin∶lotaustralin) [11]. Larvae used in the time series experiment (Figure 3D) were reared on (+)diet until they reached the third instar after which they were reared 3 weeks on (−)diet. Larvae used in the other experiments were reared on the respective diet for 2 weeks. Fifth instar larvae reared on (+)diet reached the seventh instar after 2 weeks, while seventh instar larvae reared on (−)diet for 2 weeks remained in this instar because of reduced growth [11].

### b) Dissection

In order to analyze the localization of biosynthetic gene transcripts and proteins, seventh instar *Z. filipendulae* larvae were dissected. These large last instar larvae were chosen to ease dissection and ensure high gene expression and protein levels. Following sedation in CO_2_ larvae were dissected into fat body, gonads (♀), gut, integument including nerves and muscles, labial glands and Malpighian tubules. Dissected tissues were immediately frozen in liquid nitrogen and stored at −80°C.

### c) Quantitative real-time PCR

Quantitative real-time PCR (qRT-PCR) was used to analyze the transcription of the three biosynthetic genes, and was performed in accordance with the MIQE guidelines [60]. Total RNA was extracted from whole and dissected larvae using the RNeasy Mini Kit (Qiagen, Valencia, CA, US, including digestion with the RNase-Free DNase Set (Qiagen). cDNA was synthesized using the iScript cDNA Synthesis Kit (Bio-Rad Laboratories, Hercules, CA, US). The transcript levels were analyzed by qRT-PCR, run on a Rotor-Gene Q (Qiagen) using the DyNAmo Flash SYBR Green qRT-PCR Kit (Thermo Fisher Scientific, Waltham, MA, US), with gene specific (accession number GQ915312, GQ915314 and GQ915324) and reference gene specific (targeting a homologue to the RNA polymerase II 140 kD subunit from *Drosophila melanogaster*, herein called RpII140-RA, accession number KJ192329) primers (Table S1). The reference gene was used to normalize the expression levels and enable comparison of expression of different genes from separate reactions. The qRT-PCR was run according to the following conditions: a pre-denaturation step at 95°C for 7 min, 35 cycles at 95°C for 15 s, 55°C for 30 s, 65°C for 30 s, 78°C for 15 s (respective 76°C for RpII140-RA) and subsequent acquisition, followed by a final extension step at 78°C for 15 min, and melting curve analysis from 50 to 99°C. The results were analyzed using the standard curve method with the accompanying software.

### d) RNA-Seq data generation, assembly and digital gene expression analysis

RNA-Seq was used to verify the results obtained by qRT-PCR. Total RNA was extracted from single seventh instar larvae reared on the respective host plants as described above, using the innuPREP RNA Mini Isolation Kit (Analytik Jena AG, Jena, Germany). Additional RNA purification, quantification and quality control were performed following the protocols described previously [61]. Sequencing of the two mRNA pools was performed at the Max Planck Genome Center (Cologne, Germany), RNA-Seq assays were performed on an Illumina HiSeq2500 Genome Analyzer platform, using 2×100 bp paired-end sequencing with RNA fragmented to an average of 230 bp size.

Quality control measures, including the filtering of high-quality reads based on the score value given in “fastq” files, removal of reads containing primer/adaptor sequences and trimming of read length, were performed using the CLC Genomics Workbench software 6.5 (http://www.clcbio.com). The *de novo* transcriptome assembly (TA) was performed with a total of 73 million sequence reads using the CLC Genomics Workbench software by comparing an assembly with standard settings and two additional CLC-based assemblies with different parameters, selecting the presumed optimal consensus transcriptome as described before [61]. The resulting final *de novo* TA consisted of 29,395 scaffolds larger than 250 bp with a N50 contig size of 1813 bp, an average scaffold length of 1130 bp and a maximum scaffold length of 25,190 bp. Digital gene expression analysis was carried out by using QSeq Software (DNAStar Inc., Madison, CA, US) to remap the Illumina reads obtained from the larvae onto the reference backbone and then counting the sequences to estimate expression levels. Read mapping parameters and global normalization were described previously [62].

### e) Transcript localization

To localize the transcripts of the biosynthetic genes, *in situ in tube* PCR was performed as previously described [63], with gene specific primers (Table S1) on 80 µm transverse sections prepared on a vibratome (Leica VT 1000S, Leica Microsystems, Wetzlar, Germany) from fourth to fifth instar larvae, fixed in formalin–acetic acid–alcohol (FAA) and embedded in 5% agarose. The transcript levels of earlier instars were too low for detection, while larvae of later instars could not be sectioned without disturbing the anatomy and loosing inner organs and tissues using this method. The sections were labeled by tetramethylrhodamine isothiocyanate (TRITC) and visualized using fluorescence microscopy (Leica DMR HC, filter N2.1, λ_ex_ BP 515–560 and λ_em_ LP 590). Thermocycling conditions were 98°C for 30 s followed by 35 cycles at 98°C for 10 s, 58°C for 30 s and 72°C for 20 s followed by a final extension at 72°C for 5 min.

### f) Extraction of enzymes

Total protein was extracted from intact and dissected seventh instar larvae. Following tissue disruption in liquid nitrogen, the homogenate was dissolved in 2 ml 2% dithiothreitol (DTT) in 0.2 M NaOH and centrifuged at 10,000× g for 10 min. The supernatant was recovered and precipitated with an acetone/trichloroacetic acid (80/20) solution. Following centrifugation the pellet was washed in 80% acetone, which was removed following another centrifugation step. Finally, the pellet was resuspended in 50 mM Tris-HCl buffer (pH 8.0).

### g) Western blotting

The levels of the biosynthetic proteins were analyzed using Western blotting. A crude preparation of the biosynthetic enzymes was subjected to SDS–PAGE (12% Criterion XT Bis-Tris Precast Gels, Bio-Rad), and the separated proteins were transferred to a nitrocellulose membrane (Millipore, Darmstadt, Germany) using a wet electroblotter (Bio-Rad). Following incubation with polyclonal antibodies targeting the biosynthetic enzymes (1∶1000 dilution, Figure S2) and peroxidase conjugated anti-rabbit IgG antibody produced in goat (1∶5000 dilution; Thermo Scientific Pierce, IL, US) as secondary antibody, the biosynthetic enzymes were detected using the ChemiDoc-It^2^ Imager system (Analytika Jena AG). The chemiluminescent signal was recorded using a cooled CCD camera.

### h) Immunolocalization

Immunolocalization was performed on 100 µm sections prepared on a vibratome (Leica VT 1000S) from second to third instar larvae reared on (+)diet in order to determine the localization of CYP405A2. Larvae of later instars could not be sectioned using this method. CYP405A2 was detected using polyclonal antibodies against CYP405A2 (dilution 1∶100, Figure S2) as primary antibodies and anti-rabbit IgG antibody produced in goat with fluorescein isothiocyanate (FITC) attached (dilution 1∶160) as secondary antibody (F9887; Sigma-Aldrich, MO, US) according to [64]. The localization of CYP405A2 was visualized using fluorescence microscopy (Leica DMR HC, filter N2.1, λ_ex_ BP 515–560 and λ_em_ LP 590).

### i) Statistical analysis

All statistical analyses were performed using the statistical software R [65]. Transcript data was log*_e_* and cube root transformed respectively and tested for normality using the Shapiro-Wilks normality test. After applying Fischer's exact test for equal variances, differences in gene expression between two groups were evaluated using a Mann Whitney *U* test, and differences in gene expression between more than two groups were evaluated using a Kruskal-Wallis rank sum test followed by a posthoc multiple comparison test with Bonferroni correction. Differences were considered statistically significant at *P*<0.05. All data were presented as mean ± SEM.

## Supporting Information

Figure S1 **Resin embedding and larval cross sectioning.** Fifth instar *Z. filipendulae* larvae were frozen in liquid nitrogen and stored at −80°C. Larvae were allowed to thaw and fixated in 2% paraformaldehyde in 0.2 M phosphate buffer (0.5 M Na_2_HPO_4_ and 0.23 M NaH_2_PO_4_, pH 7.0) overnight at 4°C. Following 3 times 20 min washes in phosphate buffered saline (PBS, 8 mM K_2_HPO_4_, 15.4 mM NaCl and 3.9 mM KH_2_PO_4_, pH 7.0), the samples were dehydrated 1 h each in 25, 50, 75 and 100% acetone in PBS buffer. Samples were then infiltrated with Technovit 8100 solution A (Heraeus Kulzer, Wehrheim, Germany) overnight followed by embedding in Technovit 8100 solution B according to the manufacturers protocol. After polymerization, sections (20 µm thickness) were prepared using a Reichert-Jung 2030 rotary microtome (Reichert-Jung, Germany). Cross sections were stained with Toluidine Blue and visualized using light microscopy (Leica DMR HC, Leica Microsystems).
(TIF)

Figure S2 **Generation of antibodies.** Epitope candidates were obtained using the Peptide CAD software (Agrisera AB, Vännäs, Sweden). Sequences were chosen based on localization in protein models made using the Phyre software [66], and specificity using BLAST searches against the *Z. filipendulae* transcriptome [67]. Antibodies towards CYP405A2, CYP332A3 and UGT33A1, were obtained in rabbits following injection of three pairs of haptens QNVEHRYFKTGKNI and LRRYKLSVAKEPDI, AEQLVQKIERDFVK and QVLHKYRVEPATDS, as well as KSDEVQAILKDERG and LRQVLGDDIPTLSE respectively conjugated to the 67 kDa bovine serum albumin subunit by Agrisera AB. Antibody specificity was tested by Western blots. Cross-reactive polyclonal antibodies targeting the NADPH-cytochrome P450 reductase (POR) were used as a positive control. Size of enzymes, as predicted by the ProtParam server [68]: POR – 76.37 kDa, CYP405A2 – 57.82 kDa, CYP332A3 – 58.91 kDa and UGT33A1 – 60.31 kDa.
(TIF)

Figure S3 **Tissue specific** ***de novo*** **cyanogenic glucoside biosynthetic gene expression in** ***Z. filipendulae*** **larvae.** Tissue localization of *de novo* CNglc biosynthetic genes in a representative larva determined by *in tube in situ* PCR analysis using fluorescence microscopy with 80 µm transverse sections. A) Negative control excluding primers in the PCR reaction, visualized with red light excitation and 370.4 ms exposure time. B–D) Expression of *CYP405A2*, *CYP332A3* and *UGT33A1* respectively as monitored by TRITC labeling, visualized with red light excitation and 960.8 ms exposure time. br, basal ring; cII, type II cuticular cavity; ep, epidermis; fb, fat body; hf, hair follicle; and sh, sensory hair (seta). Scale bars: 100 µm.
(TIF)

Table S1 **Primer sequences for qRT-PCR.** Primers targeting the three *de novo* CNglc biosynthetic genes and the reference gene RpII140-RA, a homologue to the RNA polymerase II 140 kD subunit from *Drosophila melanogaster*. Primer efficiency: 95–105%. T_m_, predicted melting temperature (64)/melting temperature given by melting curve; F, forward; and R, reverse.
(DOCX)

## References

[1] Duffey SS (1980) Sequestration of plant natural products by insects. Ann Rev Entomol 25: 447–477.

[2] Termonia A, Hsiao TH, Pasteels JM, Milinkovitch MC (2001) Feeding specialization and host-derived chemical defense in Chrysomeline leaf beetles did not lead to an evolutionary dead end. Proc Natl Acad Sci U S A 98: 3909–3914. 11259651 10.1073/pnas.061034598 PMC31152

[3] Engler HS, Spencer KC, Gilbert LE (2000) Insect metabolism: Preventing cyanide release from leaves. Nature 406: 144–145. 10910343 10.1038/35018159

[4] Wray V, Davis RH, Nahrstedt A (1983) Biosynthesis of cyanogenic glucosides in butterflies and moths: Incorporation of valine and isoleucine into linamarin and lotaustralin by Zygaena and Heliconius species (Lepidoptera). Z Naturforsch C 38c: 583–588.

[5] Nahrstedt A, Davis RH (1985) Biosynthesis and quantitative relationships of the cyanogenic glucosides, linamarin and lotaustralin, in genera of the Heliconiini (Insecta: Lepidoptera). Comp Biochem Physiol Pt B 82: 745–749.

[6] Nahrstedt A, Davis RH (1986) Uptake of linamarin and lotaustralin from their foodplant by larvae of *Zygaena trifolii* . Phytochemistry 25: 2299–2302.

[7] Fürstenberg-Hägg J, Zagrobelny M, Bak S (2013) Plant defense against insect herbivores. Int J Mol Sci 14: 10242–10297. 23681010 10.3390/ijms140510242 PMC3676838

[8] Lechtenberg M (2011) Cyanogenesis in higher plants and animals. *eLS*

[9] Gleadow RM, Woodrow IE (2002) Mini-review: Constraints on effectiveness of cyanogenic glycosides in herbivore defense. J Chem Ecol 28: 1301–1313. 12199497 10.1023/a:1016298100201

[10] Ballhorn DJ, Kautz S, Lieberei R (2010) Comparing responses of generalist and specialist herbivores to various cyanogenic plant features. Entomol Exp Appl 134: 245–259.

[11] Zagrobelny M, Bak S, Thorn Ekstrøm C, Olsen CE, Møller BL (2007) The cyanogenic glucoside composition of *Zygaena filipendulae* (Lepidoptera: Zygaenidae) as effected by feeding on wild-type and transgenic lotus populations with variable cyanogenic glucoside profiles. Insect Biochem Mol Biol 37: 10–18. 17175442 10.1016/j.ibmb.2006.09.008

[12] Davis RH, Nahrstedt A (1979) Linamarin and lotaustralin as the source of cyanide in *Zygaena filipendulae* I. (Lepidoptera). Comp Biochem Physiol Pt B 64: 395–397.

[13] Rammert U (1985) Untersuchungen zur wirksamkeit des wehrsekrets und des aposematischen zeichnungsmusters von *Zygaena trifolii* (Esper, 1783) auf unerfahrene Stare (Sturnus vulgaris L.). (University of Bielefeld, Bielefeld, Germany).

[14] Wiklund C, Järvi T (1982) Survival of distasteful insects after being attacked by naive birds: A reappraisal of the theory of aposematic coloration evolving through individual selection. Evolution 36: 998–1002. 28567835 10.1111/j.1558-5646.1982.tb05468.x

[15] Rammert U (1992) The reaction of birds to the larval defensive system of *Zygaena trifolii* (Esper 1783) (Lepidoptera Zygaenidae). 4th Symposium on Zygaenidae - Recent advances in burnet moth research (Lepidoptera Zygaenidae), eds Dutreix C, Naumann CM, & Tremewan WG, pp 38–52.

[16] Takos AM, Knudsen C, Lai D, Kannangara R, Mikkelsen L, et al (2011) Genomic clustering of cyanogenic glucoside biosynthetic genes aids their identification in *Lotus japonicus* and suggests the repeated evolution of this chemical defence pathway. Plant J 68: 273–286. 21707799 10.1111/j.1365-313X.2011.04685.x

[17] Jensen NB, Zagrobelny M, Hjernø K, Olsen CE, Houghton-Larsen J, et al (2011) Convergent evolution in biosynthesis of cyanogenic defence compounds in plants and insects. Nat Commun 2: 273. 21505429 10.1038/ncomms1271 PMC4354137

[18] Zagrobelny M, Bak S, Rasmussen AV, Jørgensen B, Naumann CM, et al (2004) Cyanogenic glucosides and plant-insect interactions. Phytochemistry 65: 293–306. 14751300 10.1016/j.phytochem.2003.10.016

[19] Franzl S, Naumann CM (1985) Cuticular cavities: Storage chambers for cyanoglucoside-containing defensive secretions in larvae of a Zygaenid moth. Tissue Cell 17: 267–278. 4012760 10.1016/0040-8166(85)90093-x

[20] Zagrobelny M, Bak S, Olsen CE, Møller BL (2007) Intimate roles for cyanogenic glucosides in the life cycle of *Zygaena filipendulae* (Lepidoptera, Zygaenidae). Insect Biochem Mol Biol 37: 1189–1197. 17916505 10.1016/j.ibmb.2007.07.008

[21] Zagrobelny M, Møller BL (2011) Cyanogenic glucosides in the biological warfare between plants and insects: The burnet moth-birdsfoot trefoil model system. Phytochemistry 72: 1585–1592. 21429539 10.1016/j.phytochem.2011.02.023

[22] Franzl S, Naumann CM, Nahrstedt A (1988) Cyanoglucoside storing cuticle of Zygaena larvae (Insecta, Lepidoptera). Zoomorphology 108: 183–190.

[23] Corkill L (1942) Cyanogenesis in white clover (*Trifolium repens* L.) V. The inheritance of cyanogenesis. NZ J Sci Technol B 23: 178–193.

[24] Keymer R, Ellis WM (1978) Experimental studies on plants of *Lotus corniculatis* L. from Anglesey polymorphic for cyanogenesis. Heredity 40: 189–206.

[25] Olsen KM, Kooyers NJ, Small LL (2014) Adaptive gains through repeated gene loss: parallel evolution of cyanogenesis polymorphisms in the genus *Trifolium* (Fabaceae). Philos Trans R Soc Lond B Biol Sci 369: 1471–2970. 10.1098/rstb.2013.0347 PMC4071521 24958921

[26] Armstrong HE, Armstrong EF, Horton E (1913) Herbage studies. II. - Variation in *Lotus corniculatus* and *Trifolium repens* (cyanophoric plants). Proc Roy Soc B 86: 262–269.

[27] Abbot RJ (1977) A quantitative association between soil moisture content and the frequency of the cyanogenic form of *Lotus corniculatus* L. at Birsay, Orkney. Heredity 38: 397–400.

[28] Gleadow RM, Edwards EJ, Evans JR (2009) Changes in nutritional value of cyanogenic *Trifolium repens* grown at elevated atmospheric CO2. J Chem Ecol 35: 476–478. 19352773 10.1007/s10886-009-9617-5

[29] Gebrehiwot L, Beuselinck PR (2001) Seasonal variations in hydrogen cyanide concentration of three Lotus species. Agron J 93: 603–608.

[30] Schappert PJ, Shore JS (1999) Cyanogenesis, herbivory and plant defense in *Turnera ulmifolia* on Jamaica. Ecoscience 6: 511–520.

[31] Jones DA (1988) Cyanogenesis in animal-plant interactions. Cyanide compounds in biology, eds Evered D & Harnett S (John Wiley & Sons, Chichester), pp 151–165.

[32] Arrese EL, Soulages JL (2010) Insect fat body: Energy, metabolism, and regulation. Ann Rev Entomol 55: 207–225. 19725772 10.1146/annurev-ento-112408-085356 PMC3075550

[33] Franzl S, Nahrstedt A, Naumann CM (1986) Evidence for site of biosynthesis and transport of the cyanoglucosides linamarin and lotaustralin in larvae of *Zygaena trifolii* (Insecta: Lepidoptera). J Insect Physiol 32: 705–709.

[34] Zagrobelny M, Motawie MS, Olsen CE, Pentzold S, Fürstenberg-Hägg J, et al (2014) Sequestration, tissue distribution and developmental transmission of cyanogenic glucosides in a specialist insect herbivore. Insect Biochem Mol Biol 44: 44–53. 24269868 10.1016/j.ibmb.2013.11.003

[35] Davis RH, Nahrstedt A (1985) Cyanogenesis in insects. Comprehensive insect physiology, biochemistry, pharmacology, eds Kerkut GA & Gilbert LI (Pergamon Press, Oxford), pp 635–654.

[36] Discher S, Burse A, Tolzin-Banasch K, Heinemann SH, Pasteels JM, et al (2009) A versatile transport network for sequestering and excreting plant glycosides in leaf beetles provides an evolutionary flexible defense strategy. ChemBioChem 10 (13) 2223–2229. 19623597 10.1002/cbic.200900226

[37] Kuhn J, Pettersson EM, Feld BK, Burse A, Termonia A, et al (2004) Selective transport systems mediate sequestration of plant glucosides in leaf beetles: A molecular basis for adaptation and evolution. Proc Natl Acad Sci U S A 101: 13808–13813. 15365181 10.1073/pnas.0402576101 PMC518838

[38] Oldham NJ, Veith M, Boland W, Dettner K (1996) Iridoid monoterpene biosynthesis in insects: Evidence for a *de novo* pathway occurring in the defensive glands of *Phaedon armoraciae* (Chrysomelidae) leaf beetle larvae. Naturwissenschaften 83: 470–473.

[39] Kunert M, Soe A, Bartram S, Discher S, Tolzin-Banasch K, et al (2008) *De novo* biosynthesis versus sequestration: A network of transport systems supports in iridoid producing leaf beetle larvae both modes of defense. Insect Biochem Mol Biol 38: 895–904. 18687400 10.1016/j.ibmb.2008.06.005

[40] Strauss AS, Peters S, Boland W, Burse A (2013) ABC transporter functions as a pacemaker for sequestration of plant glucosides in leaf beetles. *eLIFE* http://dx.doi.org/10.7554/eLife.01096. 10.7554/eLife.01096 PMC3843118 24302568

[41] Burse A, Schmidt A, Frick S, Kuhn J, Gershenzon J, et al (2007) Iridoid biosynthesis in Chrysomelina larvae: Fat body produces early terpenoid precursors. Insect Biochem Mol Biol 37: 255–265. 17296500 10.1016/j.ibmb.2006.11.011

[42] Burse A, Frick S, Discher S, Tolzin-Banasch K, Kirsch R, et al (2009) Always being well prepared for defense: The production of deterrents by juvenile Chrysomelina beetles (Chrysomelidae). Phytochemistry 70: 1899–1909. 19733867 10.1016/j.phytochem.2009.08.002

[43] Burse A, Frick S, Schmidt A, Buechler R, Kunert M, et al (2008) Implication of HMGR in homeostasis of sequestered and *de novo* produced precursors of the iridoid biosynthesis in leaf beetle larvae. Insect Biochem Mol Biol 38: 76–88. 18070667 10.1016/j.ibmb.2007.09.006

[44] Naumann CM, Tarmann GM, Tremewan WG (1999) The Western Palaearctic Zygaenidae: (Lepidoptera) (Apollo Books, Stenstrup, Denmark).

[45] Kahn RA, Fahrendorf T, Halkier BA, Møller BL (1999) Substrate specificity of the cytochrome P450 enzymes CYP79A1 and CYP71E1 involved in the biosynthesis of the cyanogenic glucoside dhurrin in *Sorghum bicolor* (L.) Moench. Arch Biochem Biophys 363: 9–18. 10049494 10.1006/abbi.1998.1068

[46] Davis RH, Nahrstedt A (1982) Occurrence and variation of the cyanogenic glucosides linamarin and lotaustralin in species of the zygaenidae (insecta:lepidoptera). Comp Biochem Physiol Pt B 71: 329–332.

[47] Niehuis O, Hofmann A, Naumann CM, Misof B (2007) Evolutionary history of the burnet moth genus Zygaena Fabricius, 1775 (Lepidoptera: Zygaenidae) inferred from nuclear and mitochondrial sequence data: phylogeny, host–plant association, wing pattern evolution and historical biogeography. Biol J Linn Soc 92: 501–520.

[48] Pentzold S, Zagrobelny M, Roelsgaard PS, Møller BL, Bak S (2014) The multiple strategies of an insect herbivore to overcome plant cyanogenic glucoside defence. PLOS ONE 9: e91337. 24625698 10.1371/journal.pone.0091337 PMC3953384

[49] Pasteels JM, Theuring C, Witte L, Hartmann T (2003) Sequestration and metabolism of protoxic pyrrolizidine alkaloids by larvae of the leaf beetle *Platyphora boucardi* and their transfer via pupae into defensive secretions of adults. J Chem Ecol 29: 337–355. 12737262 10.1023/a:1022629911304

[50] Davis RH, Nahrstedt A (1985) Cyanogenesis in insects. Comprehensive insect physiology, biochemistry and pharmacology, eds Kerkut GA & Gilbert LI (Pergamon Press, Oxford), Vol 11, pp 635–654.

[51] Engler-Chaouat HS, Gilbert LE (2007) *De novo* synthesis vs. sequestration: Negatively correlated metabolic traits and the evolution of host plant specialization in cyanogenic butterflies. J Chem Ecol 33: 25–42. 17151910 10.1007/s10886-006-9207-8

[52] Rowell-Rahier M, Pasteels JM (1986) Economics of chemical defense in Chrysomelinae. J Chem Ecol 12: 1189–1203. 24307055 10.1007/BF01639004

[53] Bock KW (2003) Vertebrate UDP-glucuronosyltransferases: functional and evolutionary aspects. Biochem Pharmacol 66: 691–696. 12948849 10.1016/s0006-2952(03)00296-x

[54] Ahn SJ, Vogel H, Heckel DG (2012) Comparative analysis of the UDP-glycosyltransferase multigene family in insects. Insect Biochem Mol Biol 42: 133–147. 22155036 10.1016/j.ibmb.2011.11.006

[55] Morello A, Repetto Y (1979) UDP-glucosyltransferase activity of housefly microsomal fraction. Biochem J 177: 809–812. 444205 10.1042/bj1770809 PMC1186444

[56] Real MD, Ferré J, Chapa FJ (1991) UDP-glucosyltransferase activity toward exogenous substrates in *Drosophila melanogaster* . Anal Biochem 194: 349–352. 1830726 10.1016/0003-2697(91)90239-p

[57] Ahmad SA, Hopkins TL (1992) Phenol β-glucosyltransferase and β-glucosidase activities in the tobacco hornworm larva *Manduca sexta* (L.): Properties and tissue localization. Arch Insect Biochem Physiol 21: 207–224.

[58] Ahmad SA, Hopkins TL (1993) Phenol β-glucosyltransferases in six species of insects: properties and tissue localization. Comp Biochem Physiol Pt B 104: 515–519.

[59] Luque T, Okano K, O'Reilly DR (2002) Characterization of a novel silkworm (*Bombyx mori*) phenol UDP-glucosyltransferase. Eur J Biochem 269: 819–825. 11846783 10.1046/j.0014-2956.2001.02723.x

[60] Bustin SA, Benes V, Garson JA, Hellemans J, Huggett J, et al (2009) The MIQE guidelines: Minimum information for publication of quantitative real-time PCR experiments. Clin Chem 55: 611–622. 19246619 10.1373/clinchem.2008.112797

[61] Celorio-Mancera MP, Heckel DG, Vogel H (2012) Transcriptional analysis of physiological pathways in a generalist herbivore: responses to different host plants and plant structures by the cotton bollworm, *Helicoverpa armigera* . Entomol Exp Appl 144: 123–133.

[62] Vogel H, Badapanda C, Knorr E, Vilcinskas A (2014) RNA-sequencing analysis reveals abundant developmental stage-specific and immunity-related genes in the pollen beetle *Meligethes aeneus* . Insect Mol Biol 23: 98–112. 24252113 10.1111/imb.12067

[63] Jørgensen K, Bak S, Busk PK, Sørensen C, Olsen CE, et al (2005) Cassava plants with a depleted cyanogenic glucoside content in leaves and tubers. Distribution of cyanogenic glucosides, their site of synthesis and transport, and blockage of the biosynthesis by RNA interference technology. Plant Physiol 139: 363–374. 16126856 10.1104/pp.105.065904 PMC1203385

[64] Sánchez-Pérez R, Jørgensen K, Motawia MS, Dicenta F, Lindberg Møller B (2009) Tissue and cellular localization of individual β-glycosidases using a substrate-specific sugar reducing assay. Plant J 60: 894–906. 19682295 10.1111/j.1365-313X.2009.03997.x

[65] R Development Core Team (2011) R: A language and environment for statistical computing (R Foundation for Statistical Computing, Vienna, Austria).

[66] Kelley LA, Sternberg MJE (2009) Protein structure prediction on the Web: A case study using the Phyre server. Nat Protoc 4: 363–371. 19247286 10.1038/nprot.2009.2

[67] Zagrobelny M, Scheibye-Alsing K, Jensen NB, Møller BL, Gorodkin J, et al (2009) 454 pyrosequencing based transcriptome analysis of *Zygaena filipendulae* with focus on genes involved in biosynthesis of cyanogenic glucosides. BMC Genomics 10: 574. 19954531 10.1186/1471-2164-10-574 PMC2791780

[68] Gasteiger E, Hoogland C, Gattiker A, Duvaud S, Marc R, et al. (2005) Protein identification and analysis tools on the ExPASy server. The Proteomics Protocols Handbook, Methods in Molecular Biology, ed Walker JM (Humana Press), pp 571–607.

