# Supplementary figures and images for: Chemical Defense Balanced by Sequestration and De Novo Biosynthesis in a Lepidopteran Specialist
Source: PLoS One. 2014 Oct 9;9(10):e108745. doi: 10.1371/journal.pone.0108745 (PMC4191964; doi:10.1371/journal.pone.0108745)

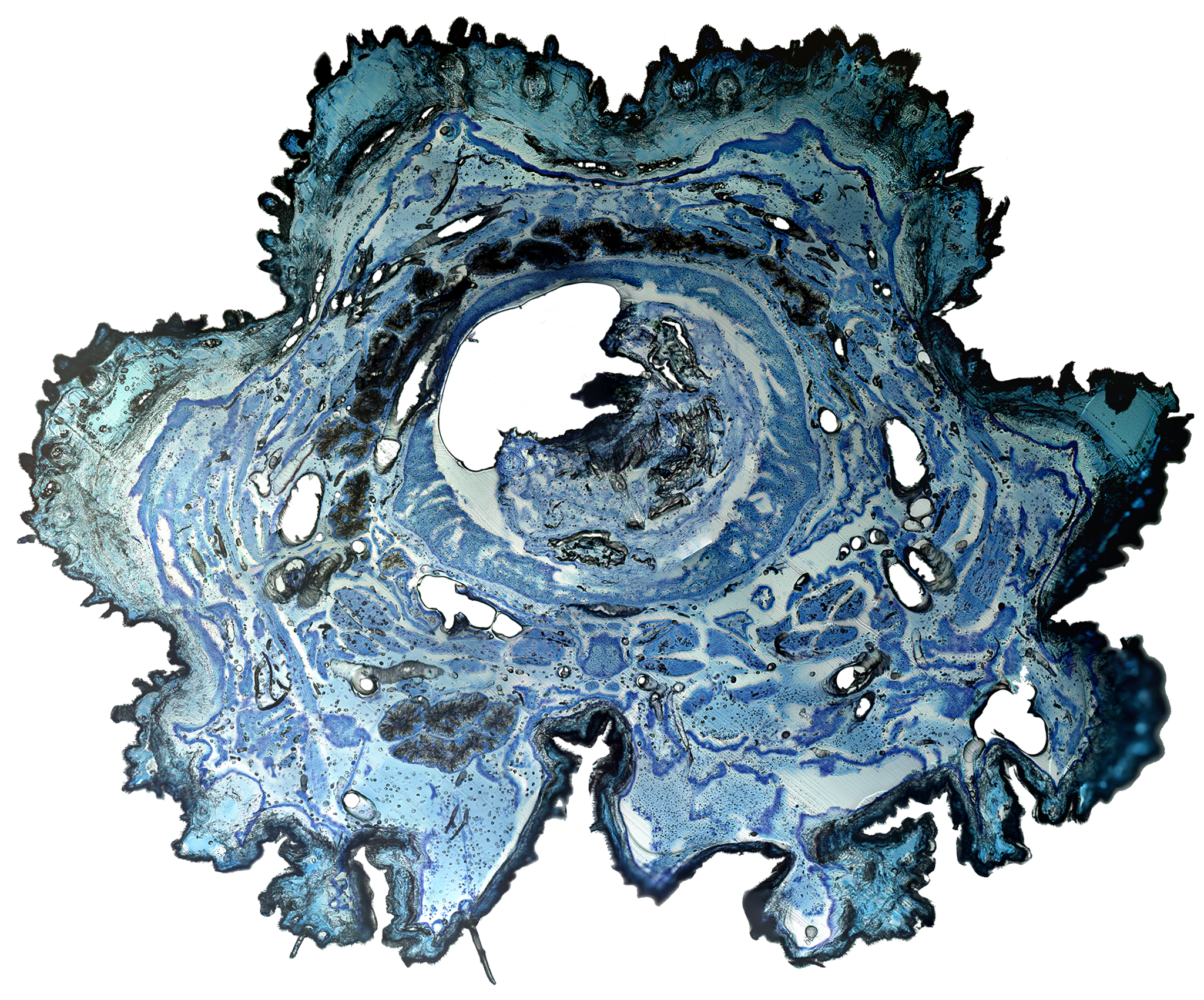

Supplement: Figure S1 — Resin embedding and larval cross sectioning. Fifth instar Z. filipendulae larvae were frozen in liquid nitrogen and stored at −80°C. Larvae were allowed to thaw and fixated in 2% paraformaldehyde in 0.2 M phosphate buffer (0.5 M Na2HPO4 and 0.23 M NaH2PO4, pH 7.0) overnight at 4°C. Following 3 times 20 min washes in phosphate buffered saline (PBS, 8 mM K2HPO4, 15.4 mM NaCl and 3.9 mM KH2PO4, pH 7.0), the samples were dehydrated 1 h each in 25, 50, 75 and 100% acetone in PBS buffer. Samples were then infiltrated with Technovit 8100 solution A (Heraeus Kulzer, Wehrheim, Germany) overnight followed by embedding in Technovit 8100 solution B according to the manufacturers protocol. After polymerization, sections (20 µm thickness) were prepared using a Reichert-Jung 2030 rotary microtome (Reichert-Jung, Germany). Cross sections were stained with Toluidine Blue and visualized using light microscopy (Leica DMR HC, Leica Microsystems). (TIF) [file pone.0108745.s001.tif]

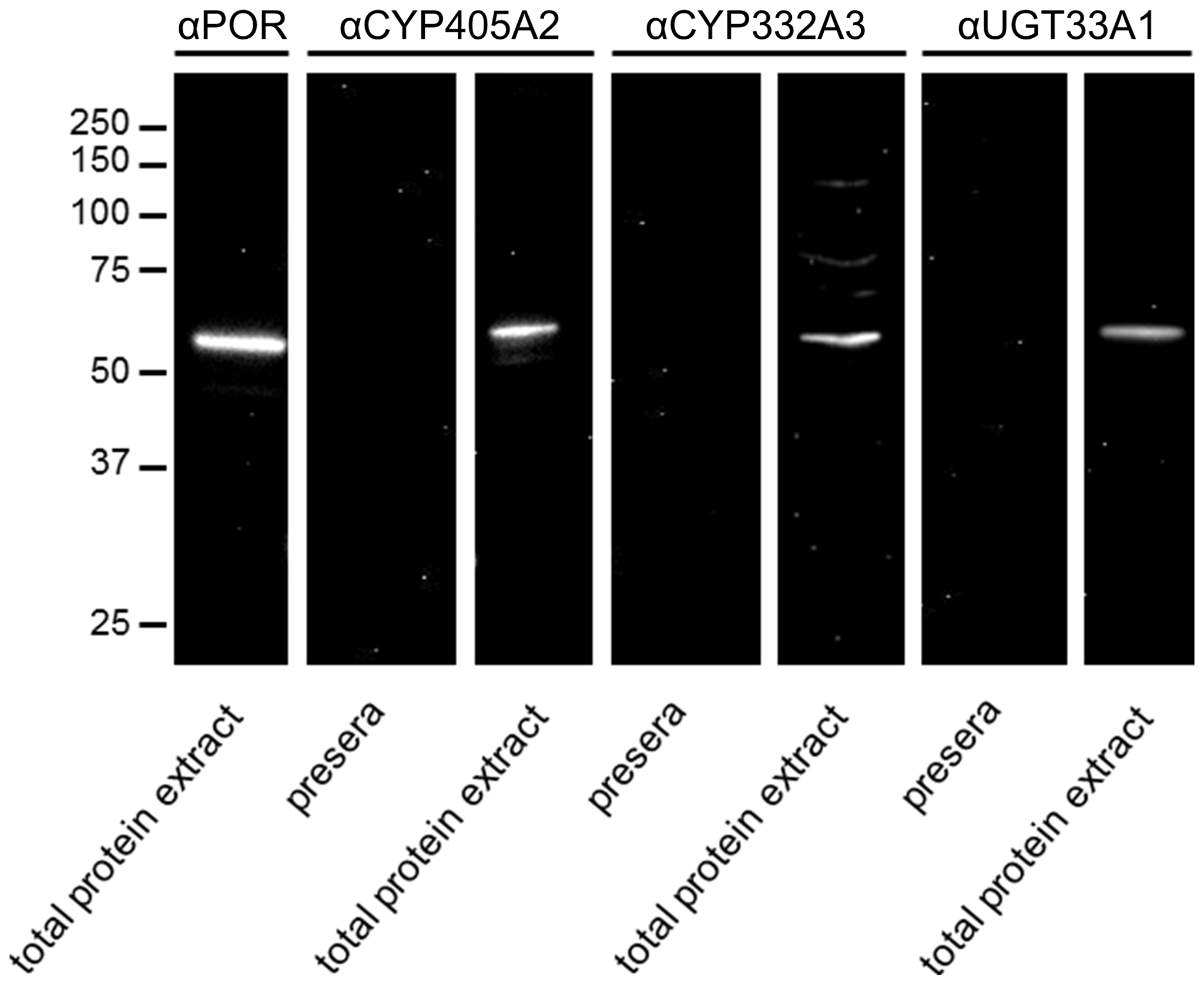

Supplement: Figure S2 — Generation of antibodies. Epitope candidates were obtained using the Peptide CAD software (Agrisera AB, Vännäs, Sweden). Sequences were chosen based on localization in protein models made using the Phyre software [66], and specificity using BLAST searches against the Z. filipendulae transcriptome [67]. Antibodies towards CYP405A2, CYP332A3 and UGT33A1, were obtained in rabbits following injection of three pairs of haptens QNVEHRYFKTGKNI and LRRYKLSVAKEPDI, AEQLVQKIERDFVK and QVLHKYRVEPATDS, as well as KSDEVQAILKDERG and LRQVLGDDIPTLSE respectively conjugated to the 67 kDa bovine serum albumin subunit by Agrisera AB. Antibody specificity was tested by Western blots. Cross-reactive polyclonal antibodies targeting the NADPH-cytochrome P450 reductase (POR) were used as a positive control. Size of enzymes, as predicted by the ProtParam server [68]: POR – 76.37 kDa, CYP405A2 – 57.82 kDa, CYP332A3 – 58.91 kDa and UGT33A1 – 60.31 kDa. (TIF) [file pone.0108745.s002.tif]

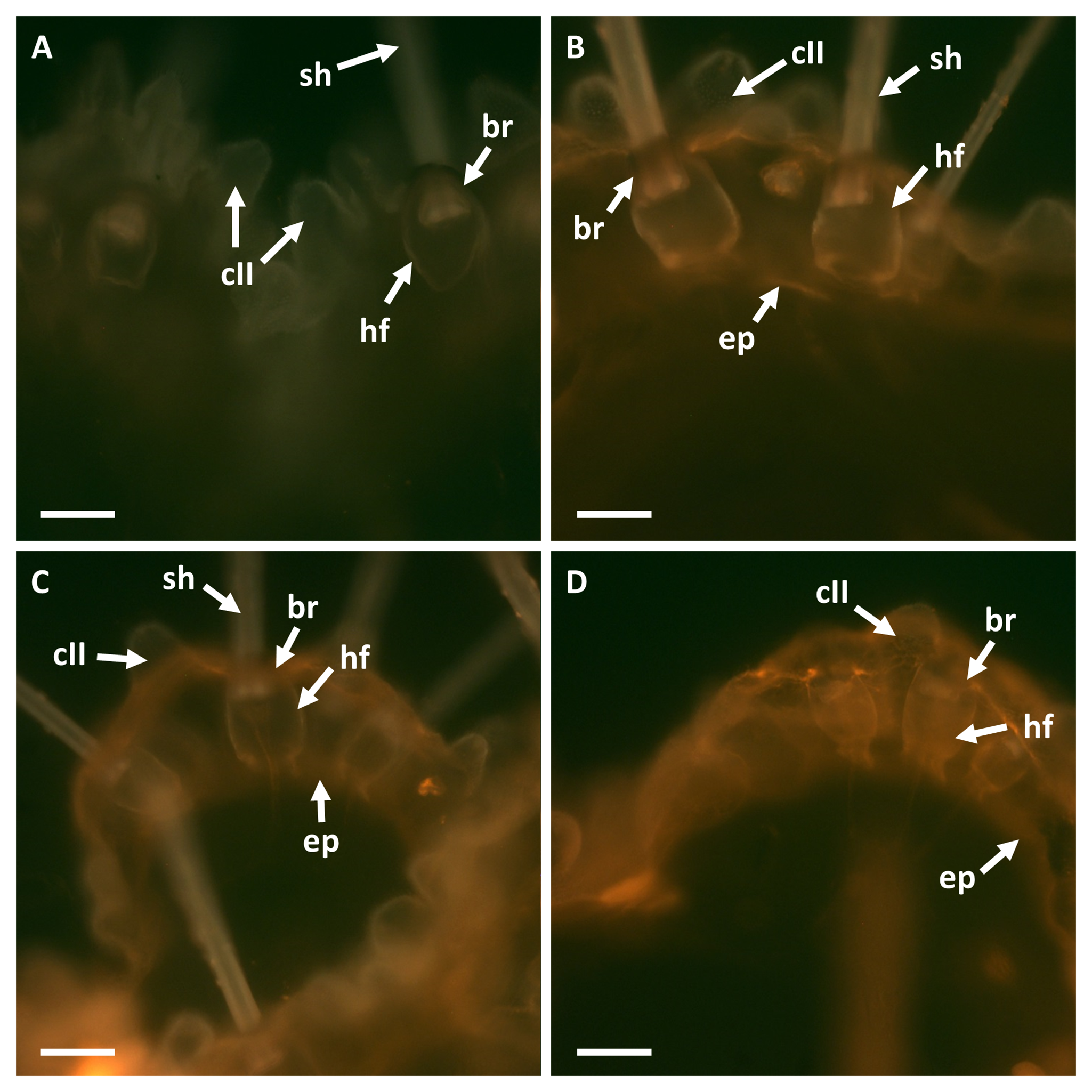

Supplement: Figure S3 — Tissue specific de novo cyanogenic glucoside biosynthetic gene expression in Z. filipendulae larvae. Tissue localization of de novo CNglc biosynthetic genes in a representative larva determined by in tube in situ PCR analysis using fluorescence microscopy with 80 µm transverse sections. A) Negative control excluding primers in the PCR reaction, visualized with red light excitation and 370.4 ms exposure time. B–D) Expression of CYP405A2, CYP332A3 and UGT33A1 respectively as monitored by TRITC labeling, visualized with red light excitation and 960.8 ms exposure time. br, basal ring; cII, type II cuticular cavity; ep, epidermis; fb, fat body; hf, hair follicle; and sh, sensory hair (seta). Scale bars: 100 µm. (TIF) [file pone.0108745.s003.tif]
