# Supplementary material for: Chemical Defense Balanced by Sequestration and De Novo Biosynthesis in a Lepidopteran Specialist
Source: PLoS One. 2014 Oct 9;9(10):e108745. doi: 10.1371/journal.pone.0108745 (PMC4191964; doi:10.1371/journal.pone.0108745)
Supplement: Table S1 — Primer sequences for qRT-PCR. Primers targeting the three de novo CNglc biosynthetic genes and the reference gene RpII140-RA, a homologue to the RNA polymerase II 140 kD subunit from Drosophila melanogaster. Primer efficiency: 95–105%. Tm, predicted melting temperature (64)/melting temperature given by melting curve; F, forward; and R, reverse. (DOCX) [file pone.0108745.s004.docx]

**Table S4. Primer sequences for qRT-PCR.** Primers targeting the three *de novo* CNglc biosynthetic genes and the reference gene RpII140-RA, a homologue to the RNA polymerase II 140 kD subunit from *Drosophila melanogaster*. Primer efficiency: 95–105 %. T_m_, predicted melting temperature ([64](#_ENREF_64)) / melting temperature given by melting curve; F, forward; and R, reverse.

| Target gene | Oligo name | Primer sequence 5’-3’ | T_m_ of primer (°C) | Length of amplicon | T_m_ of product (°C) |
| --- | --- | --- | --- | --- | --- |
|  |  |  |  |  |  |
| CYP405A2 | Zf CYP405A2 F | GGATTCAACTCCGATGAAACAT | 56.5 | 320 | 95/83 |
|  | Zf CYP405A2 R | AGCTGGTCGCAGTACTTGTT | 57.3 |  |  |
| CYP332A3 | Zf CYP332A3 F | GATGCAAAGTTCGCAGATGA | 55.3 | 316 | 95/84 |
|  | Zf CYP332A3 R | CCACAGTCACATCGTCGTTC | 59.4 |  |  |
| UGT33A1 | Zf UGT33A1 F | AAGCACCACCGAATATCACC | 57.3 | 310 | 95/84 |
|  | Zf UGT33A1 R | TAGTGGCCGAACAAAGAACC | 57.3 |  |  |
| RpII140-RA | Zf Rpol2 F | CTGTTGGCCTTGTGAAGAAT | 57.7 | 186 | 92/80 |
|  | Zf Rpol2 R | CAGGGTCTCTATGAATACCCAC | 58.5 |  |  |
